# Deprivation and chronic kidney disease—a review of the evidence

**DOI:** 10.1093/ckj/sfad028

**Published:** 2023-02-28

**Authors:** Christopher H Grant, Ehsan Salim, Jennifer S Lees, Kate I Stevens

**Affiliations:** The Glasgow Renal & Transplant Unit, Queen Elizabeth University Hospital, Govan, Glasgow, UK; College of Medical, Veterinary & Life Sciences, The University of Glasgow, Glasgow, UK; College of Medical, Veterinary & Life Sciences, The University of Glasgow, Glasgow, UK; The Glasgow Renal & Transplant Unit, Queen Elizabeth University Hospital, Govan, Glasgow, UK; College of Medical, Veterinary & Life Sciences, The University of Glasgow, Glasgow, UK; The Glasgow Renal & Transplant Unit, Queen Elizabeth University Hospital, Govan, Glasgow, UK; College of Medical, Veterinary & Life Sciences, The University of Glasgow, Glasgow, UK

**Keywords:** chronic renal failure, chronic renal insufficiency, CKD, exercise, prognosis

## Abstract

The relationship between socioeconomic deprivation and health is inequitable. Chronic kidney disease (CKD) is an archetypal disease of inequality, being more common amongst those living in deprivation. The prevalence of CKD is rising driven by an increase in lifestyle-related conditions. This narrative review describes deprivation and its association with adverse outcomes in adults with non-dialysis-dependent CKD including disease progression, end-stage kidney disease, cardiovascular disease and all-cause mortality. We explore the social determinants of health and individual lifestyle factors to address whether patients with CKD who are socioeconomically deprived have poorer outcomes than those of higher socioeconomic status. We describe whether observed differences in outcomes are associated with income, employment, educational attainment, health literacy, access to healthcare, housing, air pollution, cigarette smoking, alcohol use or aerobic exercise. The impact of socioeconomic deprivation in adults with non-dialysis-dependent CKD is complex, multi-faceted and frequently under-explored within the literature. There is evidence that patients with CKD who are socioeconomically deprived have faster disease progression, higher risk of cardiovascular disease and premature mortality. This appears to be the result of both socioeconomic and individual lifestyle factors. However, there is a paucity of studies and methodological limitations. Extrapolation of findings to different societies and healthcare systems is challenging, however, the disproportionate effect of deprivation in patients with CKD necessitates a call to action. Further empirical study is warranted to establish the true cost of deprivation in CKD to patients and societies.

## INTRODUCTION

The relationship between socioeconomic deprivation and health is both inequitable and morally unjustifiable. Deprivation can be defined by measures at both an individual and population level. The former includes household income, educational attainment and employment, whereas the latter frequently extrapolates findings from a person's postcode which may or may not reflect their individual circumstances [1]. However measured, the economic cost of inequities in health on the whole population are substantial [2].

Chronic kidney disease (CKD) can be considered to be an archetypal disease of inequality, being more common amongst those living in deprivation [3]. CKD is estimated to affect 850 million people worldwide and is predicted to be the fifth leading cause of death globally by 2050 [4, 5]. It may progress to end-stage kidney disease (ESKD) and is associated with a significantly increased risk of morbidity and mortality from cardiovascular disease (CVD) [6]. The prevalence of CKD is rising driven by an increase in lifestyle-related conditions including hypertension and diabetes [4].

This narrative review describes deprivation and its association with adverse outcomes in adults with non-dialysis-dependent CKD including disease progression, ESKD, CVD and all-cause mortality. We utilize a social determinants of health model to explore the following factors: income, employment, education, health literacy, access to healthcare, housing and air pollution. Exploring the social determinants of health and individual lifestyle factors, the following questions are addressed:

- Do patients with CKD who are socioeconomically deprived have poorer outcomes than those of higher socioeconomic status?
- Are any observed differences in health-related outcomes explained by specific socioeconomic factors?

**Figure 1:**
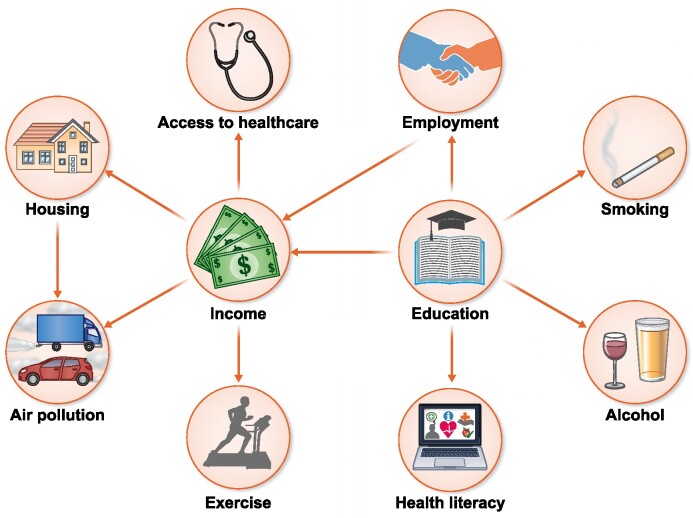
Schematic of the social determinants of health and individual lifestyle factors.

**Figure 2:**
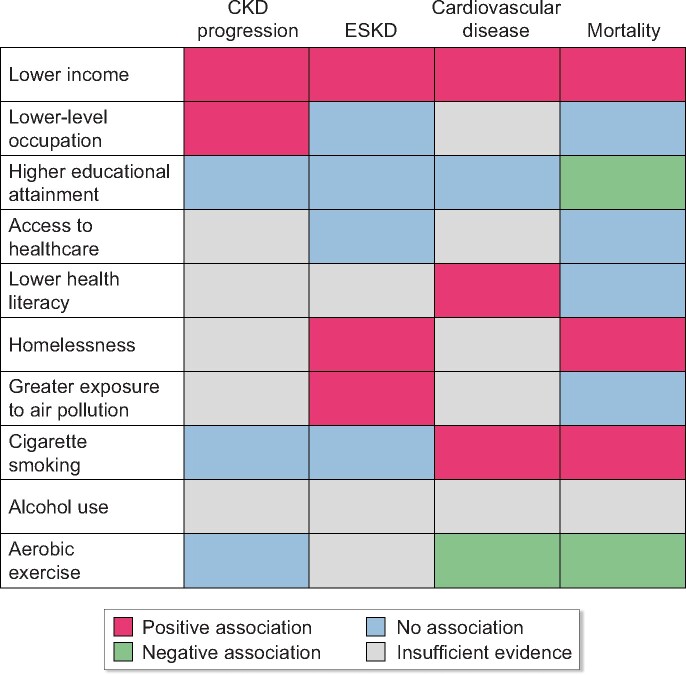
Summary of evidence assessing the social determinants of health and individual lifestyle factors with relevant outcomes in adults with CKD. *Red: positive association; green: negative association; blue: no association; grey: insufficient evidence.

**Figure 3:**
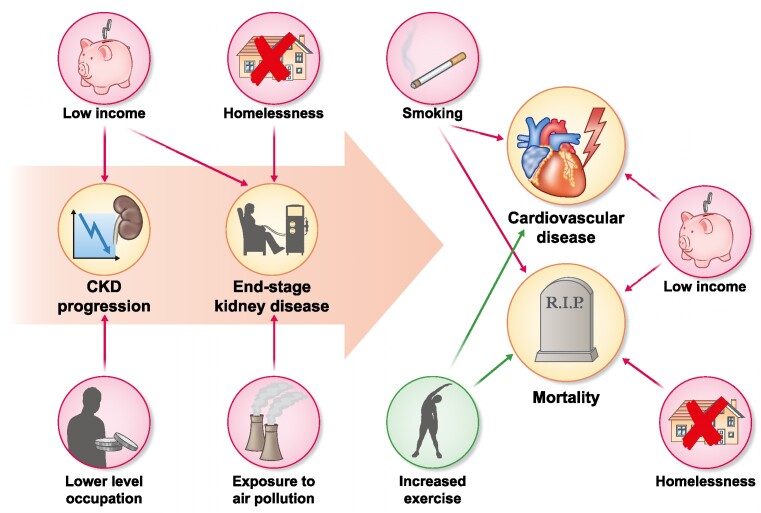
Schematic of factors associated with renal outcomes and mortality. *Red: positive association; green: negative association.

The factors examined are frequently inter-dependent, thereby making any analysis of the impact of such individual components challenging (see Fig. 1). The impact of ethnicity on adverse outcomes in CKD was deemed beyond the scope of our review and so has not been included. Table 1 and Fig. 2 summarize the strength of available evidence and primary studies included related to social determinants of health and individual lifestyle factors. Figure 3 highlights the factors associated with adverse renal outcomes and mortality.

**Table 1:** Summary of primary studies assessing the social determinants of health or individual lifestyle factors with relevant outcomes in adults with CKD.

Author [ref.] (year)	Country	Population	Relevant exposures	Outcomes
Norris *et al*. [9] (2006)	USA	CKD 2–4	Income	CVD, mortality
Alves *et al*. [10] (2010)	USA	CKD 2–4	Income, education	ESKD, CVD, mortality
Fedewa *et al*. [11] (2014)	USA	CKD 3–4	Income, education	ESKD, mortality
Hossain *et al*. [13] (2012)	UK	CKD 1–5	Education, occupation, smoking, alcohol	CKD progression, ESKD, mortality
Couchoud *et al*. [15] (2012)	France	General population	Occupation	ESKD
Morton *et al*. [18] (2016)	International	CKD 3–ESKD	Education, smoking, alcohol	CKD progression, ESKD, CVD, mortality
Winitzki *et al*. [19] (2022)	Germany	CKD 1–3	Income, education, smoking	ESKD, CVD, mortality
Bello *et al*. [21] (2012)	Canada	CKD 3–4	Income, geographical remoteness	ESKD, mortality
Jurkovitz *et al*. [22] (2013)	USA	‘At risk’ general population (HTN, DM or family history)	Education, health insurance status, smoking	ESKD, mortality
Ward *et al*. [23] (2007)	USA	Incident ESKD 2nd to lupus nephritis	Health insurance status	ESKD
Devraj *et al*. [27] (2015)	USA	CKD 1–4	Education, income, health insurance, health literacy	CKD progression
Gurgel *et al*. [32] (2021)	Netherlands	CKD 1–5	Income, education, health literacy	CVD, mortality
Hall *et al*. [33] (2012)	USA	CKD 3–5	Income, health insurance, homelessness	ESKD, mortality
Lin *et al*. [37] (2020)	Taiwan	CKD 3–4	Education, air pollution, smoking, alcohol	ESKD, mortality
Ran *et al*. [38] (2020)	Hong Kong	CKD 1–5	Income, air pollution, exercise, smoking, alcohol	Mortality
Wu *et al*. [39] (2022)	Taiwan	CKD 3b–5	Air pollution, smoking, alcohol	CKD progression
Ran *et al*. [43] (2020)	Hong Kong	CKD 1–5	Income, air pollution, exercise, smoking, alcohol	Mortality
Bundy *et al*. [46] (2018)	USA	CKD 2–4	Smoking, alcohol, exercise	CKD progression, mortality
Grams *et al*. [47] (2012)	USA	General population	Income, smoking, alcohol, exercise	CKD progression, ESKD
Ricardo *et al*. [48] (2015)	USA	CKD 2–4	Education, smoking, exercise	CKD progression, CVD
Staplin *et al*. [49] (2016)	USA	CKD 3–5 including HD or PD	Education, smoking, alcohol	CKD progression, ESKD, CVD, mortality
Hall *et al*. [50] (2016)	USA	General population	Smoking, alcohol, exercise	CKD progression
Joo *et al*. [56] (2020)	South Korea	CKD 2–4	Income, education, smoking, alcohol	CKD progression, ESKD
Robinson-Cohen *et al*. [58] (2014)	USA	CKD 1–5	Education, smoking, alcohol, exercise	ESKD, mortality
Chen *et al*. [59] (2008)	USA	CKD 3-4	Education, exercise	Mortality
Kuo *et al*. [62] (2022)	Taiwan	CKD 1–3A with proteinuria or CKD 3b–5	Exercise	ESKD, CVD, mortality
Beddhu *et al*. [63] (2009)	USA	CKD 3–5	Exercise, smoking	Mortality
Beddhu *et al*. [64] (2015)	USA	CKD 3–5	Exercise, smoking, alcohol	Mortality

DM, diabetes mellitus; HD, haemodialysis; HTN, hypertension; PD, peritoneal dialysis.

## THE SOCIAL DETERMINANTS OF HEALTH

### Income

The impact of income on health is multifaceted, affecting food security, and access to affordable housing and healthcare services. Evidence of adverse renal, cardiovascular and mortality outcomes according to income in adults with CKD is limited.

Many studies are based in the USA which does not have universal health coverage, limiting the generalizability to non-US populations. Several papers assessing renal outcomes are based on general population cohorts and are not limited to adults with CKD. These shortcomings were highlighted in two recent review articles [7, 8].

Morton *et al*. systematically reviewed the literature for evidence of a social gradient in adults with moderate to severe CKD, identifying only five studies wherein individual income was reported [7]. The sparse data related to income was effectively limited to the findings of a single randomized controlled trial from the USA.

In the African American Study of Kidney Disease and Hypertension (AASK) trial, Norris *et al*. randomized 1095 participants with CKD 2–4 to different anti-hypertensive agents and blood pressure targets to assess the impact on cardiovascular outcomes (a composite of cardiac death, myocardial infarction, stroke and heart failure). In a multivariable adjusted analysis, the authors reported that an annual income of <$15 000 at baseline was associated with a near 2-fold increased risk of adverse cardiovascular outcomes {adjusted hazard ratio (aHR) 1.94 [95% confidence interval (CI) 1.27–2.98]}. However, half of the cohort had a history of heart disease at baseline (determined by self-report, medical note review or electrocardiogram). Therefore, it is possible that these individuals had low income due to pre-existing heart disease (i.e. reverse causality) [9].

The only paper cited to have assessed the impact of income on CKD progression, all-cause and cardiovascular mortality was a subsequent study of longitudinal outcomes in AASK trial participants. The authors reported incident rate ratios (IRR) for each outcome, unadjusted for potential confounders, revealing modest increases in ESKD (4 vs 3.8/100 person years), cardiovascular composite event (3.7 vs 2.7/100 person years) and all-cause mortality (2.3 vs 2/100 person years) between those earning less than or greater than $15 000 per year, respectively. The trial was limited to patients with hypertensive nephrosclerosis, therefore results may not be generalizable to other CKD patients. Furthermore, the unadjusted nature of the analysis limits utility as the apparent relationship between income and adverse outcomes may simply reflect unmeasured confounders known to be associated with ESKD, cardiovascular events and mortality (e.g. smoking). Moreover, the possibility of comorbid illness leading to low income cannot be excluded [9, 10].

A 2018 meta-analysis highlighted seven cohort studies that assessed the impact of income on CKD progression. CKD progression was significantly associated with lower income [relative risk (RR) 1.24 (95% CI 1.12–1.37), *P* < 0.001; I2 = 66.6%, *P* = .006], however, several limitations were apparent. Six of the seven studies were based in the USA; only one adjusted for the presence of comorbid conditions; and there was variation in study recruitment, exposure and outcome definition. Furthermore, the parameters used to quantify high and low income in the meta-regression were not defined, thereby significantly limiting the utility of the findings and necessitating further study [8].

One other pertinent study was identified: Reasons for Geographic and Racial Differences in Stroke (REGARDS) was a retrospective cohort study of 2761 patients with stage 3–4 CKD in the USA, carried out between 2003 and 2007 to assess the impact of income and race on a composite outcome (incident ESKD and all-cause mortality). In a multivariable adjusted analysis, low income (annual household income <$20 000) was associated with a near one third increase in ESKD or mortality [aHR 1.32 (95% CI 1.06–1.65)]. The impact was more pronounced when comparing those in the lowest versus the highest (>$75 000) income categories [aHR 1.85 (95% CI 1.31–2.55)]. Again there were several limitations: measurement bias [defining CKD with a single glomerular filtration rate measure (GFR)], confounding (lack of adjustment for medication use) and selection bias [11].

### Employment

Employment impacts morbidity and mortality in the general population through direct and indirect effects [12]. Data on adults with CKD is comparatively sparse and of low quality with variable definitions of exposure status (e.g. employed vs unemployed or skilled vs unskilled). In the systematic review by Morton *et al*., few studies were identified that reported employment status [7]. Only one assessed its impact on cardiorenal outcomes or mortality [13].

In a retrospective cohort study from the UK, participants completed a questionnaire to identify occupation at baseline, and were followed for a median 3-year period. Occupation was categorized as skilled (47%) or unskilled (53%), and further delineated according to nine major subgroups. There was no significant association between occupation and ESKD or mortality. However, lack of variation in occupational class across deprivation quintiles suggests that both non-response and social desirability bias may have contributed to misclassification of employment status. None of the participants was reported as being unemployed and thus the findings cannot be extrapolated to a contemporary cohort of patients with CKD [13]. Unemployment has been reported to be as high as 74% in non-dialysis-dependent advanced CKD [14].

In the 2018 meta-analysis by Zeng *et al*., lower level occupation was associated with a small increase in the risk of CKD progression [RR 1.05 (95% CI 1.01–1.09), *P* = .012; I^2^ = 0.0%, *P* = .796] [8]. This was based on two observational studies: the UK study [13] and a prospective cohort study from France assessing spatial variation in kidney replacement therapy (KRT) incidence rates. The appropriateness of presenting aggregated findings in the meta-analysis is debatable: these studies were based on general population cohorts and not limited to those with CKD [8, 15].

### Educational attainment

Educational attainment may indirectly lead to poor health outcomes by affecting an individual's employability and income, and health-related behaviours [16].

In a longitudinal study of a subgroup of 14 631 Kidney Early Evaluation Programme (KEEP) participants with CKD, educational attainment was self-reported and categorized into four groups: less than high school, high school, college and professional degree attainment. In a Cox proportional hazards model adjusted for cardiovascular risk factors including hypertension and baseline estimated GFR (eGFR), educational attainment was not associated with risk of ESKD. Higher educational attainment (high school graduate or greater) was associated with lower mortality risk [aHR 0.72 (95% CI 0.62–0.83)]. Notably, the original KEEP programme was a free screening schedule which by design leads to selection bias. Medication use, which may have confounded the results, was not available. The 2097 exclusions may have been differentially excluded according to educational attainment once more contributing to selection bias [17].

In a meta-analysis, Zeng *et al*. found no association between education and CKD progression [*n* = 7 studies: RR 1.11 (95% CI 0.94–1.30) I^2^ = 71.3%, *P* = .002]. Five of the seven included studies were conducted in the USA. In a restricted analysis including two European studies, lower educational attainment was associated with higher risk of CKD progression [RR 1.23 (95% CI 1.17–1.30), I^2^ = 0.0%, *P* = .861]. However, the aggregated results were not limited to participants with CKD [8]. One study reported only unadjusted IRR for each outcome, limiting interpretability [10]. Hossain *et al*. found no association between educational attainment—classified as low (0 to 11 years of study) and normal/high (>11 years study)—and ESKD [HR 1.38 (95% CI 0.38–5.01)] or death [HR 1.15 (95% CI 0.63–2.10)], likely due to adjustment for area level socioeconomic deprivation [13].

The two most compelling contemporary studies show no independent association between educational attainment and ESKD or mortality [18, 19]. Morton *et al*. followed 9270 adults from 18 countries with moderate to severe non-dialysis and dialysis-requiring CKD and without established vascular disease for a median of 4.9 years. Among 6245 individuals with non-dialysis CKD, highest educational attainment was not associated with a composite renal outcome (ESKD or doubling of serum creatinine) or mortality following adjustment for lifestyle factors, comorbidity and CKD stage [18]. Winitzki *et al*. conducted a multi-centre, prospective cohort study in Germany, including 5095 adults with CKD over a median follow-up of 6.5 years. Low educational attainment was associated with increased risk of mortality, major adverse cardiovascular events and ESKD, but not after adjustment for baseline eGFR, albuminuria and other potential confounders. Mediation analysis identified prevalent smoking, cardiovascular disease and a number of biochemical markers that mediated the apparent association between low educational achievement and all-cause mortality [19]. It is, therefore, plausible that the relationship between educational attainment and adverse renal outcomes or mortality may be due to residual confounding or unmeasured effect modifiers.

### Access to healthcare

Access to healthcare has been defined as the timely use of health services to achieve the best possible outcomes [20]. Relevant studies exploring the impact of healthcare access in patients with CKD were limited to North America [21–23].

Geographical remoteness, as a proxy indicator of healthcare access, was not significantly associated with increased mortality in a cohort of 31 337 patients with diabetes and non-dialysis CKD in Canada. However, remote dwellers living over 100 km away from the nearest nephrology centre were more likely to progress to GFR <10 mL/min/1.73 m^2^ during follow-up [21]. Geographical remoteness may not in isolation equate to access to medical services.

In a systematic review, lack of insurance was associated with delayed referral of patients with CKD to nephrology services [24].

In a subset of KEEP study participants (*n* = 86 588), 27.8% of participants had no form of insurance. Among 12 998 participants with CKD and compared with those with private health insurance, lack of insurance was associated with all-cause mortality in CKD 1–2 [aHR1.60 (95% CI 1.13–2.26)], but not in CKD 3 or above following adjustment for traditional atherosclerotic risk factors, ethnicity and education. There was no relationship between insurance status and progression to ESKD. The findings may have been affected by the small number of participants with CKD stage 4–5 (*n* = 383). Moreover, misclassification bias likely impacted the results as a diagnosis of CKD was based upon a single serum creatinine measurement while insurance status was only measured at baseline [22].

Conversely, in a cross-sectional study of 7971 adults with ESKD attributed to lupus nephritis, those with private health insurance developed ESKD at an older age than those without. Results were not fully adjusted and thus residual confounding cannot be discounted [23].

### Health literacy

In the general population, inadequate health literacy has been associated with adverse health outcomes including mortality [25].

In people with CKD, health literacy is of particular relevance given the importance of dietary modification, medication adherence, glycaemic and blood pressure control to slow disease progression. These treatment goals require active self-management for which functional health literacy is a pre-requisite. Numerous studies have sought to capture the prevalence of inadequate health literacy in CKD with prevalence estimates varying from 18% to 63% [26–30]. There are comparatively few which examine the effect of health literacy on outcomes [31].

In a cross-sectional study of 150 adults with non-dialysis CKD from the USA, limited health literacy was associated with GFR when adjusted for multiple demographic factors, though age was a more important determinant of GFR than health literacy [27].

In the only cohort study identified, 2742 patients were followed for a median of 4.2 years to assess the impact of health literacy on self-reported CVD outcomes and mortality. After adjustment for age and sex, low health literacy was associated with CVD. This relationship was mediated by uncontrolled diabetes and obesity [32].

Poor health literacy *per se* may not be associated with adverse outcomes, but the lack of evidence denotes the need for further study.

### Housing

Adequacy of housing is inextricably linked to health. There is little empirical research on the impact of housing or homelessness on outcomes in patients with CKD; the latter reflects that such patients represent a hard-to-reach population of the most socioeconomically deprived. We identified one single-centre, retrospective cohort study of 15 343 adults with CKD in the USA who were receiving ambulatory nephrology care. Homelessness (*n* = 858; 6%) was associated with nearly one-third increased risk of ESKD or mortality after adjustment for sociodemographic, clinical and biochemical covariates [aHR 1.28 (95% CI 1.04–1.58)]. In subgroup analyses, this relationship was only evident in those without a history of substance misuse. The results may underestimate the true effect size in the population: there was selection bias in the requirement for included participants to be attending ambulatory care and hence engaging with treatment [33].

### Air pollution

Air pollution is a mix of solid, liquid and gas components suspended in atmosphere which varies within and between geographical locations. Particulate matter (PM) represents solid particles and is the major component of air pollution associated with most adverse health outcomes [34, 35]. Exposure to air pollution is often greatest among those living in areas of socioeconomic deprivation [36].

There is compelling evidence of adverse outcomes associated with air pollution in adults with CKD [37–39]. Furthermore, three recent systematic reviews suggested an association between exposure to outdoor air pollution, CKD incidence and lower GFR in the general population [40–42].

The largest individual study was a prospective cohort study of 6628 adults with CKD, conducted from 2003 to 2015 in Taiwan. Satellite-based spatiotemporal models were utilized to estimate each participant's annual PM2.5 exposure at enrolment. Increased PM2.5 exposure was independently associated with a near one-fifth increase risk of progression to KRT [*n* = 941 events; aHR 1.19 (95% CI 1.08–1.31)] with evidence of a dose–response relationship with increased exposure. No association was identified with all-cause mortality.

Misclassification bias is likely to have occurred as individuals’ outdoor activity patterns were not available to researchers and hence their recorded level of PM2.5 exposure may have been inaccurate [37]. The findings were vindicated by a recent retrospective study of adults with CKD in Taiwan, which found that after adjustment for traditional risk factors for CKD progression, PM2.5 and nitric oxide exposure was associated with increased risk of GFR decline over 2 years of follow up [39].

Two analyses of 66 820 elderly patients with CKD from Hong Kong did not demonstrate an association between PM2.5 exposure and all-cause mortality [38, 43]. However, there was a near 2-fold increase in cardiovascular mortality associated with a 4.0 mg/m^3^ increase in PM2.5 [aHR 1.97 (95% CI 1.34–2.91)]. Although the findings were based on a large cohort with 10 years of follow-up data, there were a number of unmeasured confounders—including baseline GFR, albuminuria and prevalent CVD—which may have been implicated in the causal pathway [38].

## INDIVIDUAL LIFESTYLE FACTORS

### Cigarette smoking

Cigarette smoking is more prevalent amongst those living in greater socioeconomic deprivation [44]. A recent meta-analysis suggested that cigarette smoking is an independent risk factor for incident CKD [45].

Among adults with CKD, current or past smoking has consistently been shown to be independently associated with all-cause mortality [46–49]. As in the general population, smoking is associated with cardiovascular events in CKD populations [48, 49]. A few observational studies conducted in the USA have assessed the association between smoking status and CKD progression. In a secondary analysis of the Study of Heart and Renal Protection (SHARP) trial of lipid lowering therapy in patients with CKD or on dialysis (*n* = 9270 participants; median follow-up 4 years), there was no significant difference in CKD progression (ESKD or doubling of serum creatinine) according to smoking status [49]. In the Chronic Renal Insufficiency Cohort (CRIC) study—a longitudinal study of 3939 participants followed for 5 years—the results were consistent, showing no association between smoking status and CKD progression [46].

Conversely, the Jackson Heart Study included 5301 African American volunteers in a prospective cohort study to assess adverse renal outcomes between a baseline (2000–04) and follow-up visit (2009–12). Current smokers had a higher IRR for eGFR decline compared with never or past smokers [IRR 1.83 (95% CI 1.31–2.56)]. There was also a dose-dependent increase in CKD progression events depending upon the number of cigarettes smoked [50]. The conflicting results among these three studies may reflect differences in the outcome definitions: Hall *et al*. defined their CKD progression event as a drop in GFR of 30% between study visits whereas SHARP and CRIC studies specified GFR decline of 50% or ESKD as the outcomes of interest [46, 49, 50].

### Alcohol use

Alcohol use is not more prevalent in those living in deprivation, however, the adverse effects of consumption are disproportionately experienced [51]. Excessive alcohol use is associated with hypertension, liver disease and malignancy in the general population. There is extensive evidence to suggest that alcohol use, particularly light or moderate consumption, is associated with a reduction in the incidence of CKD compared with abstinence amongst the general population [52–55]. However, few studies have quantified the impact of alcohol use on adverse outcomes in patients with established CKD. Two observational studies from the USA and South Korea reached different conclusions regarding the impact of alcohol use on CKD progression [46, 56]. In 3933 participants from the CRIC study, there was no difference in CKD progression events between those who reported persistent alcohol use compared with non-drinkers. There was a modest reduction in all-cause mortality amongst drinkers [aHR 0.73 (95% CI 0.58–0.91)], which may be explained by the exclusion of patients with liver cirrhosis and the measurement of alcohol consumption in a binary rather than graded manner [46]. Conversely, in the Korean Cohort Study for Outcome in Patients with CKD (KNOW-CKD), Joo *et al*. followed 1883 patients with CKD for a renal composite endpoint (>50% eGFR decline or ESKD) according to alcohol use. Over a median follow-up of 3.0 years, regular [HR 2.20 (95% CI 1.38–3.46)] and occasional binge [HR 2.00 (95% CI 1.33–2.98)] drinking were associated with a 2-fold increase in CKD progression [56].

The paucity and inconsistency of evidence necessitates further study.

### Aerobic exercise

The benefits of aerobic exercise are well established [57]. In adults with CKD, socioeconomic deprivation is associated with reduced physical activity [58, 59]. People living in deprivation often have poorer access to green spaces and fewer opportunities for regular physical activity. The effect of this is compounded in patients with CKD who are frequently overweight and experience greater comorbidity and physical deconditioning with disease progression [60].

There is little evidence to suggest that aerobic exercise directly influences disease progression in people with CKD. A systematic review and meta-analysis of 15 trials found that aerobic exercise had no significant impact on GFR trajectory [61]. Similar results were reported in the CRIC observational study [48].

There is evidence that aerobic exercise reduces both adverse cardiovascular outcomes and all-cause mortality [62–65]. In the largest observational study including 906 adults with CKD over 8.8 years of follow-up, a higher level of self-reported physical activity was associated with lower hazards of all-cause mortality in insufficiently active [HR 0.58 (95% CI 0.42–0.79)] and active [HR 0.44 (95% CI 0.33–0.58)] compared with inactive participants [63].

## SOCIOECONOMIC DEPRIVATION AND OUTCOMES IN CHRONIC KIDNEY DISEASE

Although many of the studies mentioned adjusted for sex in their analyses, none stratified their results according to sex. It is difficult to comment upon any interaction between sex and socioeconomic deprivation.

### Progressive CKD and ESKD

Studies of socioeconomic disadvantage in adults with CKD have yielded conflicting results with regards to adverse renal outcomes. A 2018 meta-analysis of observational studies assessing indices of socioeconomic status and CKD progression included 12 cohort studies that predominantly assessed income or education at baseline. Pooled analyses demonstrated that lower combined socioeconomic status was associated with a one-third increase risk of CKD progression [RR 1.39 (95% CI 1.09–1.79), *P* = .009; I^2^ = 74.2%, *P* = .009]. However, the results varied according to geographic location. There was also considerable heterogeneity in the classification of socioeconomic indicators, definition of CKD and subject recruitment. Furthermore, many studies assessed socioeconomic exposures only at baseline rather than accounting for the potential effect of social mobility during follow-up, potentially leading to misclassification bias [8].

The findings were supported by a retrospective cohort study from the UK, which also found that area level socioeconomic deprivation was associated with CKD progression, but not ESKD. The authors followed 918 of 1427 patients attending regular renal clinic follow up for a median of 3 years. On multivariate logistic regression, compared with the least deprived quintile (Q5) those living in greatest deprivation (Q1) were at a 2-fold risk of both CKD progression, i.e. >2 mL/min/1.73 m^2^/year loss [aHR 2.17 (95% CI 1.14–4.51)], and rapid progression, i.e. >5 mL/min/1.73 m^2^/year loss [aHR 2.07 (95% CI 1.05–4.36)] [13].

The results were inconsistent with another retrospective cohort study from Italy that examined 715 consecutive patients with CKD undergoing nephrology care for at least 12 months. Patients were followed for a median of 10 years for adverse renal and cardiovascular outcomes and mortality after 1 year of evidence-based, goal-directed therapy. Participants were delineated according to an area measure of socioeconomic attributes determined by census including education, employment, home ownership, single parent family and overcrowding. Among this cohort of older-aged adults with predominantly mild–moderate CKD, deprivation was not associated with CKD progression or ESKD. Although compelling, the findings may have been affected by both survival and measurement bias. Patients were required to attend for 12 months of renal follow-up to be included which may have failed to capture those less likely to attend, amongst whom deprivation may have been more prevalent. Socioeconomic status was deduced from the 2001 census despite follow-up to 2018. Misclassification may have occurred whereby participants relocated or achieved greater material wealth over the course of follow-up. Both of these biases would have under estimated the effect of disadvantage on adverse outcomes [66].

In the only prospective cohort study identified, Solbu *et al*. followed 2950 unselected adults with CKD from first attendance at a renal clinic in the UK for a median of under 2 years. In adjusted analyses, residing in an area of greatest socioeconomic deprivation was not associated with ESKD [67].

Patients living in areas of greater socioeconomic deprivation present later to nephrology services. In a cross sectional study of 1657 patients with CKD in the UK, living in the lowest quintile of socioeconomic status was independently associated with a lower eGFR at first nephrology assessment [68]. In a systematic review, lower socioeconomic status was associated with late referral to nephrology services [24]. Late presentation in CKD is associated with adverse outcomes, including mortality [69]. In a population in France with broadly equitable access to healthcare provision, the incidence of KRT for ESKD has been shown to increase along a social gradient according to a multi-dimensional index of deprivation [70].

Patients with CKD from socioeconomically deprived areas have also been shown to be at greater risk of hospitalization than more affluent counterparts even after adjusting for individual lifestyle factors and comorbidity [71].

### Cardiovascular outcomes and mortality

Socioeconomic deprivation is associated with all-cause mortality in CKD.

In Scotland, living in the most deprived area was independently associated with all-cause mortality [aHR 1.60 (95% CI 1.10–2.34)]. However, the findings must be interpreted cautiously: cause of death was unknown in 33% of cases. Furthermore, the authors were unable to capture data pertaining to baseline comorbidity and smoking which may have confounded any relationship between deprivation and mortality [67]. A larger retrospective cohort study of 13 400 participants with CKD in the USA found that socioeconomic advantage—assessed by a composite of income, employment, education, marital status and substance misuse—was positively associated with decreased mortality [aHR 0.88 (95% CI 0.86–0.89)] [72]. There was no adjustment for access to treatment by means of healthcare insurance coverage and so extrapolating these results to other countries with universal health coverage is difficult. Neither study above found an association with cardiovascular mortality [66, 67].

In a 2016 systematic review, low educational attainment, income and lack of home ownership were significantly associated with adverse cardiovascular outcomes and increased mortality [7].

The only discordant finding for the impact of socioeconomic deprivation on all-cause mortality was in the UK study by Hossain *et al*.; however, non-response bias may have contributed to an under estimate of adverse outcomes, with those living in areas of socioeconomic deprivation less likely to participate [13].

## CONCLUSION

CKD can be considered to be an archetypal disease of deprivation, with inequitable differences in outcomes. The impact of socioeconomic deprivation in adults with non-dialysis-dependent CKD is complex, multi-faceted and frequently under-explored within the literature.

This review summarizes the evidence in this patient cohort with reference to the wider structural influences on health including selected individual lifestyle risk factors. There is evidence that patients with non-dialysis-dependent CKD who are socioeconomically deprived have faster disease progression and higher risk of both CVD and premature mortality. This seems to be the result of both socioeconomic and individual lifestyle factors. However, delineating the relatively contribution of individual socioeconomic factors that mediate this complex relationship is challenging given the inter-dependent nature of the social determinants of health (Fig. 1). The difficulty in isolating any one factor (e.g. education from employment) is likely to at least in part explain the significant paucity of studies in many areas.

The major gaps within the literature to date pertain to relative contributions of income, employment, healthcare access, health literacy, housing, air pollution and alcohol use (Fig. 2). In many cases, these gaps simply reflect a lack of empirical study. This may represent a perception amongst researchers that the generalizability of findings from any one centre or region to other societies with different and complex healthcare systems is of dubious importance. Undoubtedly, a limitation within the evidence to date is the number of studies from countries without universal health coverage. Diagnosis and management of CKD requires laboratory testing and hence (timely) access to medical services. Other key limitations include: the retrospective nature of many studies, relatively short follow-up, variable definitions of socioeconomic exposures and significant under-representation of individuals at the highest risk of poor outcomes (e.g. advanced CKD, homeless populations, etc.). The latter is likely to represent that these patients are often the hardest to reach and hence capture in health research. They are, however, likely to be in the greatest need of targeted interventions to improve health. Our narrative review is important as it highlights both the scarcity of research and low-quality evidence underpinning our understanding of the complex relationship between deprivation and CKD.

It is clear that the disproportionate effect of deprivation in patients with CKD necessitates a call to action: that patients from more deprived areas present later and with lower GFR is of major concern and highlights a cohort that could be identified for early intervention, yielding health and economic benefits. Further empirical study is warranted to establish the true cost of deprivation in CKD to patients, societies and healthcare systems.
